# Transcriptome sequencing of field pea and faba bean for discovery and validation of SSR genetic markers

**DOI:** 10.1186/1471-2164-13-104

**Published:** 2012-03-20

**Authors:** Sukhjiwan Kaur, Luke W Pembleton, Noel OI Cogan, Keith W Savin, Tony Leonforte, Jeffrey Paull, Michael Materne, John W Forster

**Affiliations:** 1 Department of Primary Industries, Biosciences Research Division, Victorian AgriBiosciences Centre, 1 Park Drive, La Trobe University Research and Development Park, Bundoora, Victoria 3083, Australia; 2 Department of Primary Industries, Biosciences Research Division, Grains Innovation Park, Horsham, Victoria 3401, Australia; 3 La Trobe University, Bundoora, Victoria 3086, Australia; 4 School of Agriculture, Food and Wine, The University of Adelaide, Waite Campus, Glen Osmond, South Australia 5064, Australia

## Abstract

**Background:**

Field pea (*Pisum sativum *L.) and faba bean (*Vicia faba *L.) are cool-season grain legume species that provide rich sources of food for humans and fodder for livestock. To date, both species have been relative 'genomic orphans' due to limited availability of genetic and genomic information. A significant enrichment of genomic resources is consequently required in order to understand the genetic architecture of important agronomic traits, and to support germplasm enhancement, genetic diversity, population structure and demographic studies.

**Results:**

cDNA samples obtained from various tissue types of specific field pea and faba bean genotypes were sequenced using 454 Roche GS FLX Titanium technology. A total of 720,324 and 304,680 reads for field pea and faba bean, respectively, were *de novo *assembled to generate sets of 70,682 and 60,440 unigenes. Consensus sequences were compared against the genome of the model legume species *Medicago truncatula *Gaertn., as well as that of the more distantly related, but better-characterised genome of *Arabidopsis thaliana *L.. In comparison to *M. truncatula *coding sequences, 11,737 and 10,179 unique hits were obtained from field pea and faba bean. Totals of 22,057 field pea and 18,052 faba bean unigenes were subsequently annotated from GenBank. Comparison to the genome of soybean (*Glycine max *L.) resulted in 19,451 unique hits for field pea and 16,497 unique hits for faba bean, corresponding to c. 35% and 30% of the known gene space, respectively. Simple sequence repeat (SSR)-containing expressed sequence tags (ESTs) were identified from consensus sequences, and totals of 2,397 and 802 primer pairs were designed for field pea and faba bean. Subsets of 96 EST-SSR markers were screened for validation across modest panels of field pea and faba bean cultivars, as well as related non-domesticated species. For field pea, 86 primer pairs successfully obtained amplification products from one or more template genotypes, of which 59% revealed polymorphism between 6 genotypes. In the case of faba bean, 81 primer pairs displayed successful amplification, of which 48% detected polymorphism.

**Conclusions:**

The generation of EST datasets for field pea and faba bean has permitted effective unigene identification and functional sequence annotation. EST-SSR loci were detected at incidences of 14-17%, permitting design of comprehensive sets of primer pairs. The subsets from these primer pairs proved highly useful for polymorphism detection within *Pisum *and *Vicia *germplasm.

## Background

The Fabaceae (Leguminosae) is the third largest angiosperm family, containing c. 18,000 species attributed to 650 genera [1-3]. Legumes provide major benefits to cropping systems and the environment, due to the ability to perform symbiotic nitrogen fixation. In comparison to cereals, for which a broad range of genetic and genomic resources are available, genomic databases for legumes are generally still underdeveloped. However, recent advances in sequencing and genotyping technologies offer the opportunity to rapidly ameliorate the status of given species at relatively low cost [4]. Major efforts are currently being directed towards the development of species-specific genomic tools and datasets. As an example, the whole genome sequence of soybean, a warm-season grain legume, has recently been determined http://www.phytozome.net/soybean[5].

Cool-season food legumes within the Hologalegina clade of the Fabaceae sub-family Papilionoideae, which includes lentil, chickpea, field pea and faba bean (pulses), are important food and fodder crops, especially in developing countries such as those of the Indian sub-continent [6]. These species are important components of farming systems across Western Asia, the Middle East, North Africa, the Indian sub-continent, North America and Australia. In Australia, pulses are sown over c. 2 million hectares and produce c. 2.5 million tonnes of grain with a commodity value of over AU$ 675 million [7]. Despite close phylogenetic relationships, pulse species vary considerably in aspects of biology such as genome size, fundamental chromosome number, ploidy level, and degree of reproductive self-compatibility. The genome size of chickpea is relatively small (c. 700 Mb), but pulses of the Vicieae tribe (lentil, pea and faba bean) exhibit much larger genome sizes (in the range from 4-13 Gb). Recently, generation of large-scale lentil transcriptome data by our group has substantially increased the volume of publicly available genomic data for this species [8]. Similar strategies have been pursued for field pea and faba bean in the current study.

Field pea, which is the third most globally important grain legume crop (at 5.5 million hectares per year) after soybean and common bean (*Phaseolus vulgaris *L.), is a self-pollinating diploid (2n = 2x = 14) species with a genome size of c. 5 Gbp [1]. Various studies have been performed to determine the genetic basis of multiple phenotypic traits in field pea [9-11] and to quantify diversity between different pea cultivars [12-16]. Recently, a comprehensive transcriptome analysis of field pea has been performed using second-generation sequencing technologies [17] that will contribute significantly to the enrichment of genomics resources for field pea. In contrast, faba bean has not been widely adopted on a global basis. In terms of cultivation area, this species ranks fourth among the cool-season food legumes (at 2.6 million hectares per year) after field pea, chickpea and lentil http://faostat.fao.org. Faba bean has been traditionally cultivated in the Mediterranean basin, the Nile valley, Ethiopia, Central and East Asia, Latin America, Northern Europe, North America and Australia [18]. Faba bean is a diploid taxon (2n = 2x = 12), and exhibits facultative cross-pollination at frequencies ranging from 4-84%. The nuclear genome size of faba bean is one of the largest yet described among crop legumes, at c. 13 Gb. Formal genetic analysis of faba bean, such as through genetic linkage mapping and identification of quantitative trait loci (QTLs), has so far been hindered by these aspects of biology [19].

Conventional breeding methods based on phenotypic assessment are currently in use for breeding line selection in field pea and faba bean. Such methods are logistically demanding and time-consuming, especially for traits that require specific biotic or abiotic challenges, such as resistance to individual diseases. In addition to this, when breeding for types eaten as immature seed, quality testing adds considerable complexity to the relevant programs. There is consequently a major requirement for species-specific molecular genetic markers and derived linkage maps for field pea and faba bean, to enable germplasm advancement through genomics-assisted selection.

Current publicly available genetic and genomic tools for field pea and faba bean are limited in extent [20-23], comprising 18,552 and 5,253 ESTs, respectively that are available in Genbank. In addition to this, a recently sequenced *Pisum sativum *transcriptome generated a total of 81,449 unigenes that are also available for download as a fully annotated fasta format [17]. Second-generation DNA sequencing systems such as the Roche 454 massively-parallel pyrosequencing platform are capable of rapidly producing species-specific genomic resources to address these short-comings. This system can generate 4-6 × 10^8 ^bp from each run, with individual read lengths of 400-500 bp [24], and is suitable for *de novo *sequencing of small genomes [25], whole genome resequencing [26], SNP detection [27], and in particular, sequencing of transcriptomes [28].

ESTs obtained from the latter activity provide valuable resources for gene discovery, large-scale expression analysis, improved genome annotation, elucidation of phylogenetic relationships and facilitation of breeding programs for both plants and animals through provision of SSR and single nucleotide polymorphism (SNP) genetic markers [29]. SSR loci have been widely used for improvement of a range of crop species [30]. Only a limited number of SSRs are available in public domain for field pea and faba bean, creating an incentive for further discovery and validation. In comparison with genomic DNA-derived SSRs, those located in ESTs are functionally associated with genic regions, and support potential diagnostic genetic marker development [31-34].

This study describes the development, *de novo *assembly and gene annotation of a transcriptome dataset derived from cDNA samples obtained from several tissues at various stages of development of multiple field pea and faba bean genotypes. Clustering and annotation to generate a unigene set has permitted computational identification of SSR loci, and the design and evaluation of a set of EST-SSR marker-directed primer pairs.

## Materials and methods

### Plant material

Seeds of field pea were obtained from the Australian Temperate Field Crops Collection (ATFCC) held at the Department of Primary Industries, Horsham, Victoria, Australia. Faba bean seeds were obtained from the Australian faba bean breeding program at The University of Adelaide, South Australia, Australia. Three to four seeds from each variety of field pea (Parafield, Yarrum, Kaspa, 96-286*) and faba bean (Icarus, Ascot) were selected based on the criteria of genetic diversity and significant agronomic variation, and were sown into commercial potting mix. These genotypes were also potential parents for the genetic mapping populations of field pea and faba bean, to be used to dissect various traits of interest. Germinated plantlets were grown to maturity under glasshouse conditions with natural light at the Department of Primary Industries, Bundoora, Victoria, Australia. Selected plant tissues were harvested for RNA isolation from plants at various stages of development, including leaf (young and mature), stem, flowers, immature pods, mature pods and immature seeds. A total of 4-8 seeds were also germinated in Petri dishes in order to provide material for harvest of seedling root and shoot samples. All of the vegetative plant tissues (leaf and stem) were pooled for RNA isolation and designated LS (leaf/stem) tissue. All of the reproductive organs including flowers, immature pods, mature pods and immature seeds were also pooled for RNA isolation and designated FS (flower/seed) tissue. The seedling-derived root (RG) and shoot (SG) samples were used separately for RNA isolation.

### RNA isolation and cDNA preparation

Total RNA isolation and cDNA synthesis were performed as described in an equivalent study performed for lentil [8].

### EST sequence generation, assembly and annotation

cDNAs obtained from the four distinct RNA pools (LS, FS, RG and SG) were combined in equimolar ratio before proceeding to GS FLX library preparation. Approximately 5 μg of bulked cDNA was sheared by nebulisation at 206 kPa for 2-4 min. The GS FLX Titanium shotgun libraries were constructed following manufacturer's instructions (Roche Diagnostics, Castle Hill, NSW, Australia). The ssDNA libraries were quantified using real-time quantitative PCR. Finally, emulsion (em) PCR was performed using the Lib-L emPCR protocol (Roche Diagnostics, Castle Hill, NSW, Australia). The enriched beads obtained as a result of em-PCR were loaded onto picotitre plates for sequencing. All of the pooled cDNA libraries obtained from different genotypes of field pea and faba bean were separately sequenced on individual quarters of picotitre plates.

All sequence reads generated from different genotypes were *de novo *assembled using the Next *Gene *software (Softgenetics, State College, Pennsylvania, USA). The adaptor and primer sequences were removed prior to the assembly using the 'trimming' function (trim sequences with 100% similarity to the primer/adaptor sequence). *De novo *assembly was performed using the Greedy algorithm and error correction condensation. The Greedy algorithm searches for maximum overlap between reads and extends the overlap to form large contigs and is recommended for 454 reads or reads with average read length > 70 bp. The error correction condensation tool functions by dividing sequence reads in which homopolymers are found and at least 16 bases intervene between the homopolymer runs. These shorter reads were termed keywords, and comparison of keywords between reads allowed the correct determination of the bases at the end of each keyword. Sequence reads that contain variations of low frequency were then corrected.

Assembled contig outputs were deposited in the Transcriptome Shotgun Assembly (TSA) of GenBank (field pea; JR950756-JR964200 and faba bean; JR964201-JR970413). Contigs and singletons were compared against the *M. truncatula *(Mt 3.0), *A. thaliana *(TAIR 9 CDS [coding sequences]), *G. max *(Glyma 1.0) and *P. sativum *[17] transcriptome databases using BLASTN [35] with a threshold E value of 10^-10^. Both field pea and faba bean unigene sets were also BLASTN analysed against respective EST and nucleotide sequences publicly available in GenBank. BLASTN analysis was also performed in the non-redundant database of GenBank using the tBLASTX algorithm to derive putative annotations of the unigene set. Gene ontology (GO) terms were assigned to unigenes that showed hits against the *Arabidopsis thaliana *database using the 'Gene Ontology at TAIR' tool.

### Discovery of EST-SSRs, primer design and marker validation

Detection of EST-SSR loci and primer pair design was performed using the Batch Primer3 software http://probes.pw.usda.gov/cgi-bin/batchprimer3/batchprimer3.cgi. The parameters were designed for identification of perfect di-, tri-, tetra-, penta-, and hexanucleotide motifs with minimum of repeat numbers of 6, 4, 3, 3, and 3, respectively. Primer design parameters were set as follows: length range = 18 to 23 nucleotides with 21 as optimum; PCR product size range = 100 to 400 bp; optimum annealing temperature = 55°C; and GC content 40-60%, with 50% as optimum.

Genomic DNA was extracted from target plant genotypes for EST-SSR marker validation using the DNeasy^® ^96 Plant Kit (QIAGEN), following the manufacturer's instructions. Frozen leaf tissue from each genotype was used for each extraction and ground using a Mixer Mill 300 (Retsch^®^, Rheinische Straße, Haan, Germany). DNA was resuspended in 50 μl of water and dilutions were performed to obtain a final concentration of 10 ng/μl, followed by storage at -20°C. A collection of randomly selected EST-SSR primer pairs were validated experimentally, forward primers being synthesised with addition of a bacteriophage M13-matching sequence, to enable fluorescent tail addition through the PCR amplification process

[36]. PCR conditions included a hot-start at 95°C for 10 minutes, followed by 10 cycles of 94°C for 30 s, 60-50°C for 30 s and 72°C for 30 s, followed by 25 cycles of 94°C for 30 s, 50°C for 30 s and 72°C for 30 s and a final elongation step of 72°C for 10 min. PCR products were separated using an ABI3730xl (Applied Biosystems, Foster City, California, USA) according to manufacturer's instructions with the addition of the ABI GeneScan LIZ500 size standard and amplification product sizes were determined using the GeneMapper^® ^v3.7 software (Applied Biosystems).

## Results

### EST sequencing and *de novo *assembly

**Figure 1:**
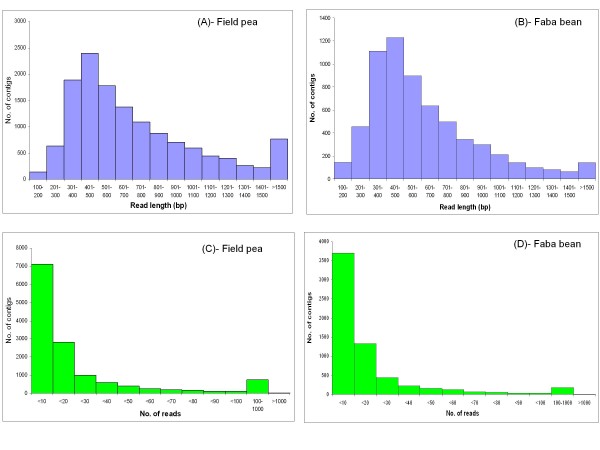
**Frequency histograms showing the distribution of number of contigs versus function of read length (A, B)/no. of reads (C, D) in field pea and faba bean, respectively**.

A total of 720,324 and 304,680 reads were generated from a range of sampled tissues from 4 field pea genotypes and 2 faba bean genotypes, respectively, using the GS FLX Titanium chemistry. In addition to adaptor/primer sequence trimming, strings of 30-40 nucleotides from both the 5'- and 3'-termini of each sequence read were removed in order to generate high confidence data. Table 1 summarises the sequence output data for each species. After clustering and assembly, a total of 13,602 contigs and 86,476 singletons were obtained from field pea, representing a total of 100,078 unigenes (Additional files 1 and 2). In case of faba bean, a total of 86,027 of unigenes were obtained, comprising 6,370 contigs and 79,657 singletons (Additional files 3 and 4). The unigene sets were then further assessed for quality based on read length, and any remnant sequences less than 100 bp were excluded from further analysis, leaving a total of 13,583 contigs and 57,099 singletons (field pea) and 6,351 contigs and 54,089 singletons (faba bean). In field pea, the length of contigs ranged from 100 bp to 6587 bp, with an average of 719 bp, while for faba bean, contig length ranged from 104 bp to 3923 bp with an average of 615 bp. Average contig coverage was 13.8 fold (ranging from 1.20-fold to 21846.96-fold) for field pea and 8.9 fold (ranging from 1.26 fold to 2884.64 fold) for faba bean. The number of reads per contig for field pea varied between 2 and 57,215, with an average of 41, and the corresponding values for faba bean were between 2 and 16,713 with an average of 25 (Table 2). Distributions of read length and number of reads per contig are shown in Figure 1. The number of contigs with read length less than 200 bp was minimal (1% in field pea and 2.2% in faba bean). Most of the contigs were longer than 0.5 kb (62.7% in field pea and 53.7% in faba bean). In both species, the majority of the contigs were derived from less than 10 reads (Figure 1C, D). A total of 5.7% field pea contigs and 2.9% faba bean contigs were composed of more than 100 reads.

**Table 1:** Summary of GS FLX sequencing outputs (total number of reads, cumulative sequence output, median read length, number of reads used for assembly)

Species	Total number of reads generated	Cumulative sequence (Mbp)	Median read length (bp)	Number of reads used for assembly
Field pea	720,324	261	389	687,200
Faba bean	304,680	83	277	248,448

**Table 2:** Summary of data on contig assemblies for field pea and faba bean

Number of reads per contig	Number of contigs		Percentage of total contigs per read number class	
	
	Field pea	Faba bean	Field pea	Faba bean
2	239	71	1.8	1.1
3	642	247	4.7	3.9
4	1504	768	11.1	12.1
5	1348	747	9.9	11.8
6	1129	645	8.3	10.2
7	897	494	6.6	7.8
8	785	402	5.8	6.3
9	579	325	4.3	5.1
10	514	267	3.8	4.2
11-15	1573	772	11.6	12.2
16-20	873	343	6.4	5.4
21-25	512	231	3.8	3.6
26-30	409	184	3.0	2.9
31-35	310	129	2.3	2.0
36-40	269	83	2.0	1.3
41-45	221	84	1.6	1.3
46-50	173	78	1.3	1.2
> 50	1606	481	11.8	7.6

**Figure 2:**
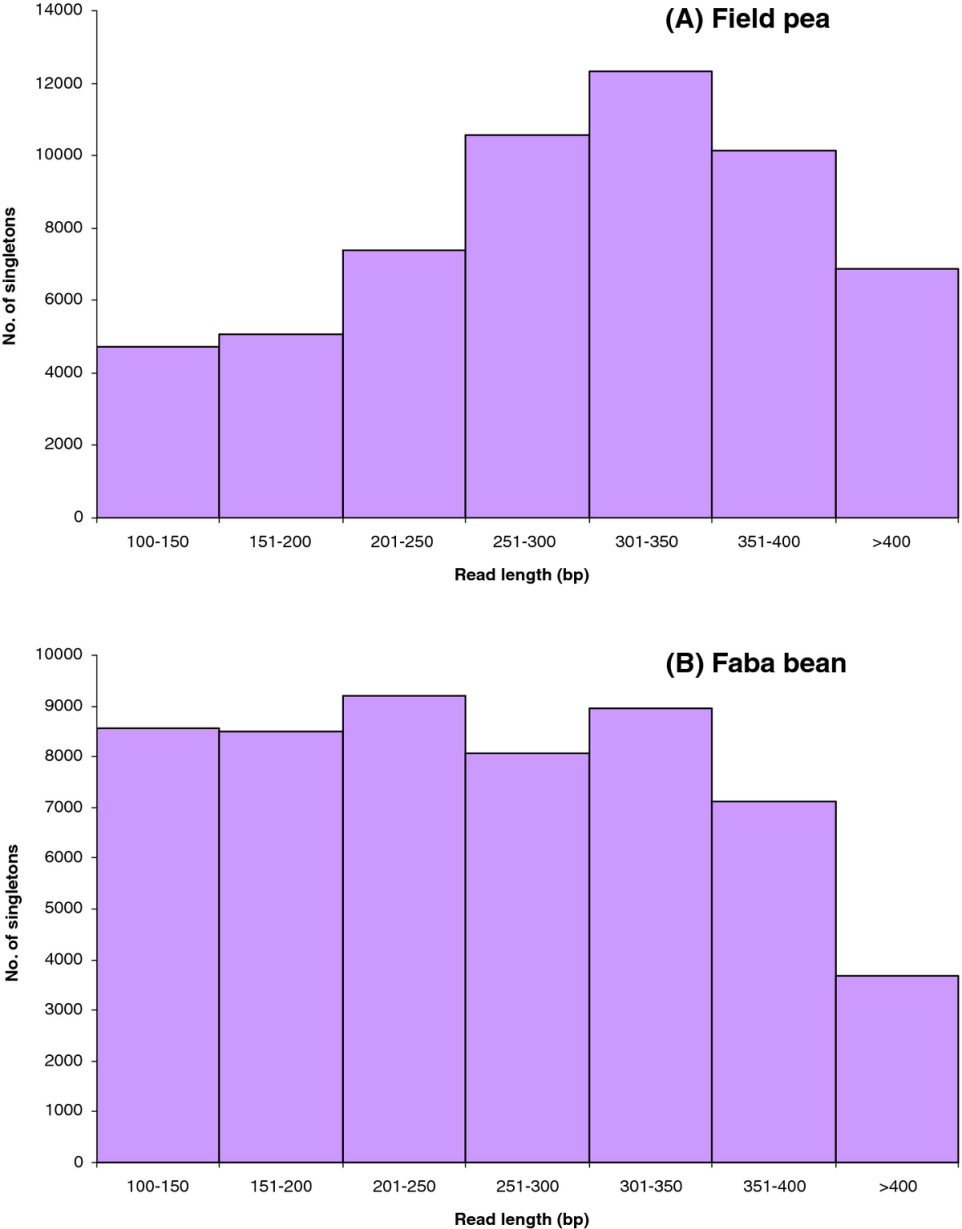
**Frequency histograms indicating the distribution of number of singletons as a function of read length in field pea (A) and faba bean (B)**.

The length of singletons varied from 100-540 bp (field pea) and 100-537 bp (faba bean). For field pea, the largest proportion of the singletons (21.6%) varied from 301-350 bp, while for faba bean, the majority of singletons (17%) varied from 201-250 bp (Figure 2).

### Gene annotation

Since *M. truncatula *is the model legume species that is most closely related to field pea and faba bean, consensus sequences from all contigs and singletons were preferentially compared to *Medicago *coding sequences. In case of field pea, a total of 11,737 unique matches were obtained (6,224 contigs and 5,513 singletons) (Additional file 5). The unigene set was also compared against the nr database of GenBank. A total of 9,101 contigs and 13,194 singletons (22,295 unigenes) obtained matches at E < 10^-10^. Any query sequences that revealed a highest-ranking match against a non-plant species were removed from the list, leaving a total of 22,057 unique hits (Additional file 6 sheet 'final'). Finally, all of the consensus sequences were compared against the *A. thaliana *database. A total of 6,156 unique matches were obtained, consisting of 3,668 contigs and 2,488 singletons (Additional file 7).

The faba bean unigene set was also compared with the *M. truncatula *genome and a total of 10,179 hits were obtained (3,246 contigs and 6,933 singletons) at E < 10^-10 ^(Additional file 8). The unigene set was subsequently compared to the nr database of GenBank, resulting in 18,244 unique hits composed of 4,508 contigs and 13,736 singletons. Any sequence that matched a non-plant database entry was removed from the list, resulting in 18,052 unique hits (4,668 contigs and 13,584 singletons) (Additional file 9, sheet 'final'). The unigene set was also compared to the *A. thaliana database *at a threshold value of E < 10^-10 ^(Additional file 10), and a total of 4,883 hits were obtained, consisting of 1,948 contigs and 2,935 singletons. Finally, the field pea and faba bean unigene sets were also compared against the *G. max *EST sequence database that identified 19,451 unique matches for field pea and 16,497 for faba bean (Additional file 11). 'The contigs and singletons obtained from field pea in the current study were also compared against the unigene set generated from transcriptome analysis of field pea performed by Franssen et al. (2011) and as a result, a total of 45,161 overlapping hits were identified (10,832 contigs [24%] and 34,329 singletons [76%]) (Additional file 12). In some instances, more than one contig revealed hits to the same gene, which may be due to origin of more than one contig or singleton from a single gene due either to non-overlapping sequence reads or high levels of sequence error in a single read. This process has also demonstrated the benefits obtained from comparison between two complementary studies.

All of the ESTs and nucleotide sequences currently available in GenBank for field pea and faba bean were also downloaded on the local server to perform BLASTN searches against field pea and faba bean contigs and singletons obtained from the current study. In case of field pea, a total of 2,764 EST and 77,431 nucleotide sequences obtained from Genbank showed significant hits against unigene set generated in the current study (corresponding to 2,244 and 31,624 unique hits, respectively) (Additional file 13, sheets 1-2). For faba bean, a total of 549 ESTs (222 unique matches against faba bean unigene set) and 3,684 nucleotides (1,277 unique matches against faba bean unigene set) were found be common between Genbank and transcriptome data generated from the current study (Additional file 13, sheets 3-4).

**Figure 3:**
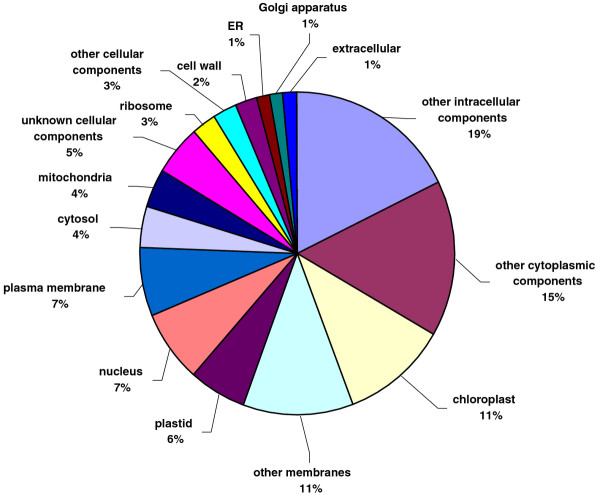
**Pie-chart representation of GO annotation results from field pea consensus sequences for cellular process components**.

**Figure 4:**
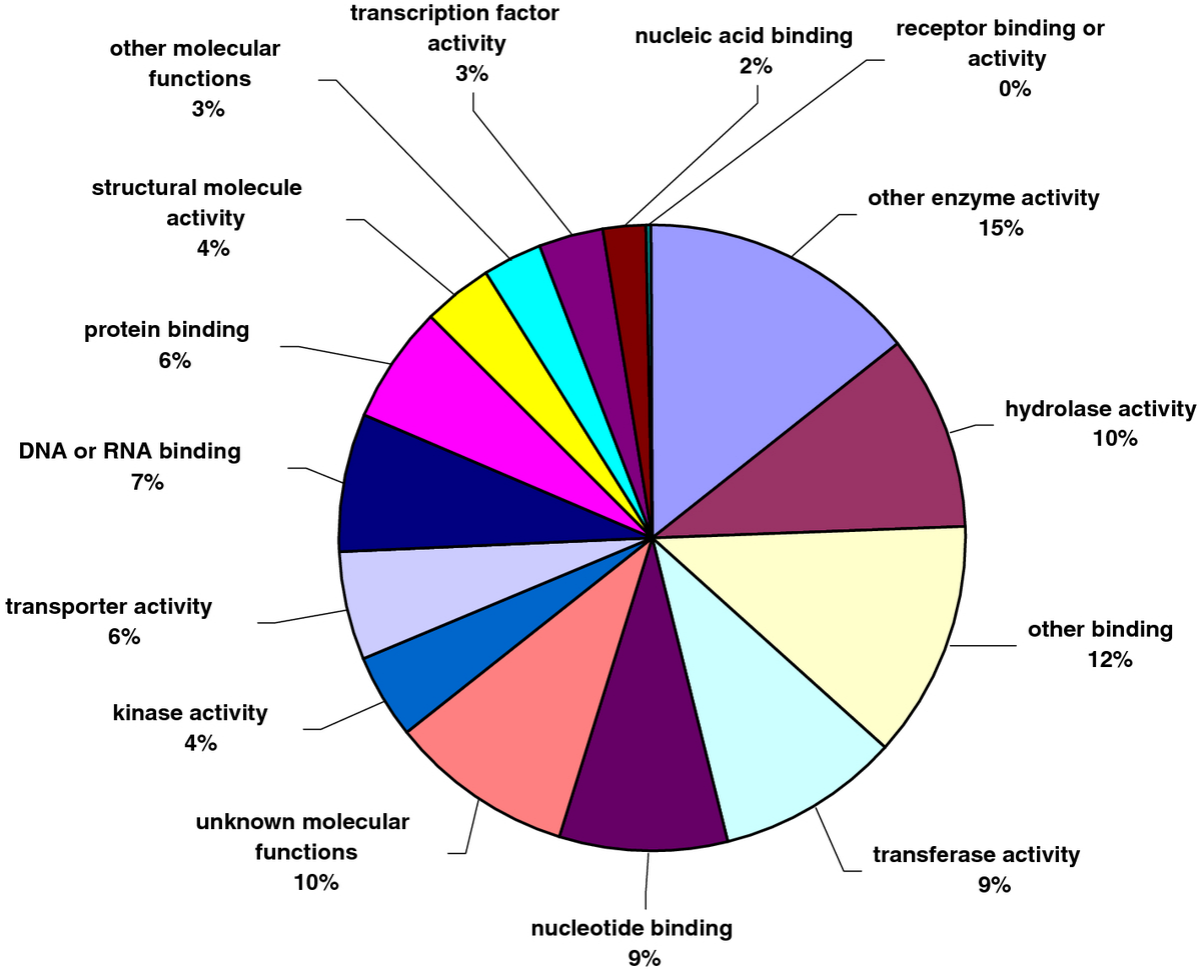
**Pie-chart representation of GO annotation results from field pea consensus sequences for molecular process components**.

**Figure 5:**
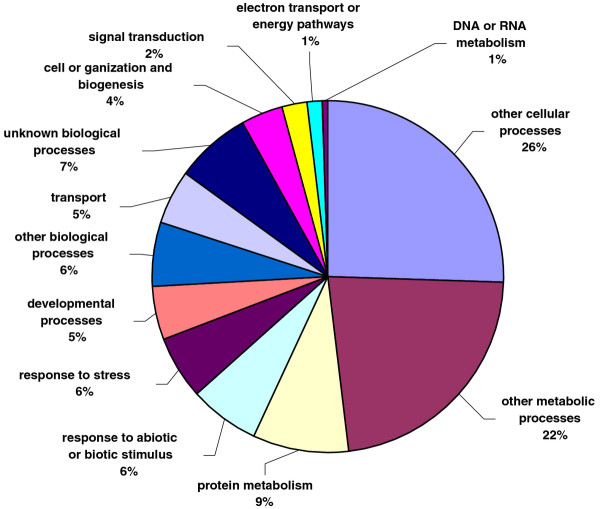
**Pie-chart representation of GO annotation results from field pea consensus sequences for biological process components**.

**Figure 6:**
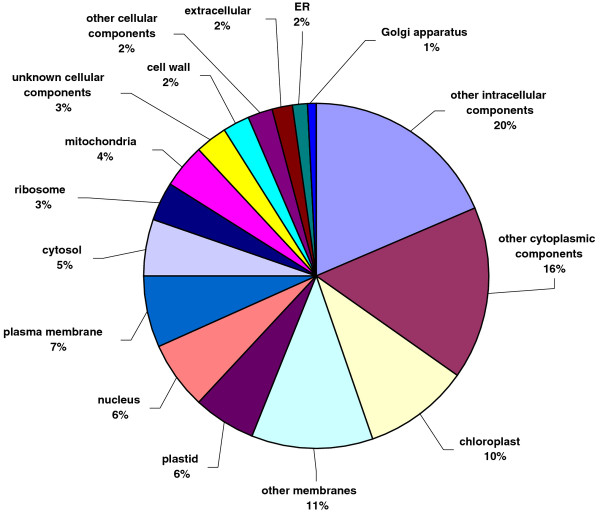
**Pie-chart representation of GO annotation results from faba bean consensus sequences for cellular process components**.

**Figure 7:**
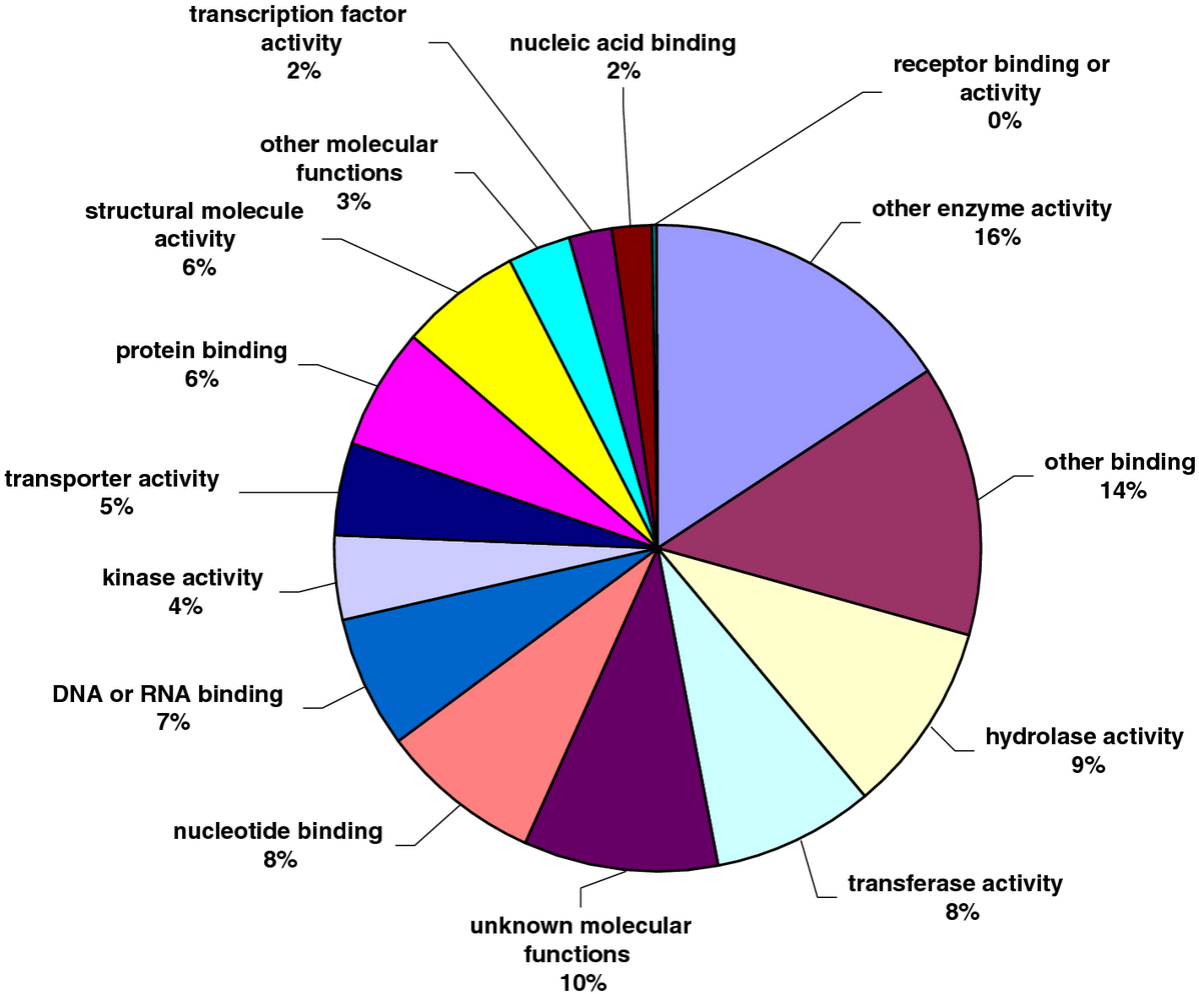
**Pie-chart representation of GO annotation results from faba bean consensus sequences for molecular process components**.

**Figure 8:**
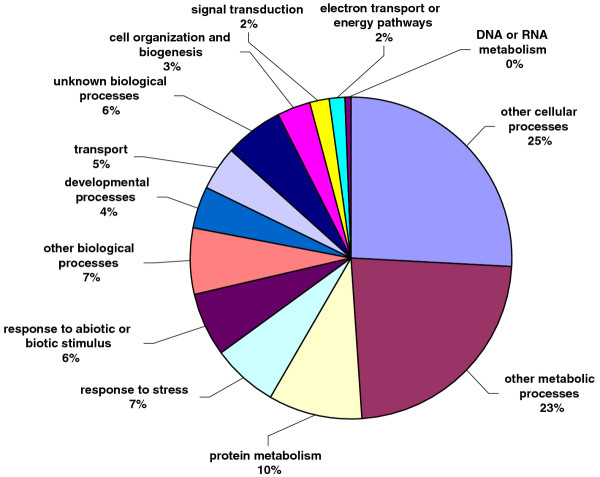
**Pie-chart representation of GO annotation results from faba bean consensus sequences for biological process components**.

All unique matches obtained from field pea and faba bean contigs by comparison against the *A. thaliana *database were annotated and GO terms were further assigned. For field pea, a total of 22,068 gene counts and 30,739 annotation counts were obtained, while for faba bean, these corresponding values were 11,869 gene counts and 17,075 annotation counts. Proportions of each unigene set attributed to major functional categories were determined (Figures 3, 4, 5, 6, 7, 8). In case of field pea, the intracellular component category of the cellular component classification class contributed the largest proportion of all annotations (19%), followed by the cytoplasmic component (15%), chloroplast component (11%), membrane component (11%), nuclear component and plasma membrane component (7%) categories. Other components such as plastid, cytosol, mitochondria, ER, golgi apparatus, cell wall, ribosome and extracellular components were represented at proportions less than 5% of total (Figure 3). Among the molecular function classification class, the enzyme activity, binding activity, hydrolase activity, transferase activity, molecular function and nucleotide binding categories included the majority of detected matches (Figure 4). In the biological processes classification class, cellular (26%) and metabolic processes (22%) constituted the major categories, followed by protein metabolism (9%) and unknown biological processes (7%), (Figure 5). Similar results were obtained for faba bean. In the cellular component classification class, the major contributors were intracellular and cytoplasmic components (20% and 16% respectively) (Figure 6). The enzyme activity (16%), binding activity (14%) and unknown molecular functions (10%) categories contributed the most in molecular function classification class (Figure 7) while among the biological processes classification class, cellular and metabolic processes (25% and 23% respectively) constituted the major categories (Figure 8).

### EST-SSR discovery

In field pea, EST-SSR discovery was performed based on analysis of assembled contig templates, of which 2,345 (17%) contained at least one repetitive motif. A total of 2,932 distinct loci were identified, 588 template contigs containing at least two SSR loci eligible for primer pair design. A total of 2,397 SSR primer pairs were designed from these 2,932 distinct loci (Additional file 14, sheet Fieldpea). In the case of faba bean, a total of 1,097 distinct loci were identified in 914 of 6,351 assembled contigs (14%), from which 802 SSR primer pairs were designed (Additional file 14 sheet Fababean). Incidences of different repeat types were determined (Table 3), the most abundant being trinucleotide arrays for both field pea (1,383; 57.7%) and faba bean (495; 61.7%). Frequencies for each array type according to repeat unit number were also evaluated (Table 3), the most common class being n = 4 (43.3% for field pea and 48.6% for faba bean).

**Table 3:** Frequencies of different SSR repeat motif types observed in field pea and faba bean

SSR motif length	Repeat unit number											
	**Species**	3	4	5	6	7	8	9	10	> 10	Total	%
Dinucleotide	Field pea	0	0	0	87	34	14	8	4	3	150	6.3
	Faba bean	0	0	0	19	19	13	4	1	0	56	7.0
Trinucleotide	Field pea	0	952	270	111	35	10	4	0	1	1383	57.7
	Faba bean	0	370	86	17	15	4	1	1	1	495	61.7
Tetranucleotide	Field pea	432	45	5	1	0	0	0	0	0	483	20.2
	Faba bean	124	7	1	1	0	0	0	0	0	133	16.6
Pentanucleotide	Field pea	147	17	3	0	1	0	0	0	0	168	7.0
	Faba bean	41	6	1	0	0	0	0	0	0	48	6.0
Hexanucleotide	Field pea	180	24	6	0	2	1	0	0	0	213	8.9
	Faba bean	59	7	4	0	0	0	0	0	0	70	8.7
Total	Field pea	759	1038	284	199	72	25	12	4	4	2397	
	Faba bean	224	390	92	37	34	17	5	2	1	802	
%	Field pea	31.7	43.3	11.8	8.3	3.0	1.0	0.5	0.2	0.2		
%	Faba bean	27.9	48.6	11.5	4.6	4.2	2.1	0.6	0.2	0.1		

### Validation of EST-SSR assays

A subset of 96 EST-SSR primer pairs each from field pea and faba bean data sets were selected for validation of marker assay performance. For field pea, a total of 86 (90%) successfully obtained amplification products from one or more template genotypes, of which 40 (46.5%) revealed polymorphism between 5 genotypes of field pea. Inclusion of a template sample from the non-domesticated species *PS3689 *(wild type landrace accession of *Pisum sativum *from Afghanistan) permitted polymorphism detection by 11 additional primer pairs (an increase to 59.3% of total) (Additional file 15, sheet Fieldpea). For faba bean, 81 primer pairs (84%) exhibited successful amplification, of which 24 detected polymorphic (29.6%) between cultivated *V. faba *genotypes (Icarus and Ascot). When the non-domesticated *V. faba *genotype ACC118 was included in the analysis, polymorphism rate increased to 48% (Additional file 15, sheet Fababean).

## Discussion

### EST assembly and gene annotation

The increasing capacity of DNA sequencing technologies has permitted substantial increases in genomic resource availability for several legume crops that had been previously underdeveloped. Recently, large-scale transcriptome characterisation using the GS FLX platform has been performed for both lentil and pigeonpea [8,37]. This technology can deliver large amounts of data at considerably lower costs as compared to traditional sequencing methods, and so provides an effective means to expedite analysis of less-studied species [31]. In the present study, equivalent approaches have been applied to the two Vicieae species, field pea and faba bean, in order to develop a transcribed sequence database and to identify and validate EST-SSRs.

GS FLX sequencing has been shown to ineffectively process homopolymer regions that are longer than 8 bp in length [38]. Therefore, poly(A) tails at mRNA termini may present major challenges, and result in under-representation of the 3'-ends of transcripts. In the present study, the problem was resolved through use of a modified primer with an interrupted polyd(T) tail. This contributed to an increase in the output of the total number of sequenced fragments by c. 6% (data not shown). A number of other transcriptome studies have used the same approach to overcome the homopolymer sequencing problems [39,40].

Prior to sequencing, normalisation of the cDNA samples obtained from leaf and stem tissues was performed in order to increase the sequencing efficiency of rare transcripts. The normalisation process helps to reduce over -sampling of abundant transcripts that are presentin high quantities, hence increasing confidence of detecting a larger proportion of rare transcripts. Preliminary experiments indicated that normalisation of leaf/stem cDNA could increase the possibility of detecting rare transcripts by c. 10% (unpublished data). Similar approaches have been applied to detect rare transcripts in lentil, *M. truncatula, Artemisia annua *and greenhouse whitefly [8,41-43].

The average contig lengths for the target species in this study are comparable to those observed in other studies (*Pisum sativum*, 454 bp [17], *Pinus contorta*, 500 bp [44]; lentil, 770 bp [8]; sweet potato, 790 bp [45]; mungbean, 843 bp [19]). A large proportion of the reads assembled into contigs in case of field pea (87%), which is comparable to the values observed in other studies (Glanville fritillary butterfly, 91% [46]; *Eucalyptus grandis*, 88% [47]; *Acropora millepora *larvae, 90% [48]). In contrast, a relatively smaller proportion (65%) of reads from faba bean assembled into contigs, resulting in lower length and depth as compared to the data derived from field pea. This may be due to the fact that the sequencing output for faba bean was comparatively smaller than that of field pea. Similar results have been observed in other studies [45]. As a result of *de novo *assembly, a large number of singletons were obtained both for field pea (86,476) and faba bean (79,657), also as observed for other species [17,42,44,48]. Although some singletons may arise as contaminating sequences or artefacts, the majority probably originate from transcripts expressed at low levels, and were consequently retained in the dataset. Many singleton sequences (15% for field pea and 17% for faba bean) exhibited high read quality due to matching of protein-encoding genes in the existing genic databases, and hence provide valuable sources of information. The remaining singletons could have resulted from various reasons such as incompleteness of known databases, sequencing errors, short read lengths leading to difficulty in assembly etc. [8,31].

BLAST searches against databases of model plant species provided annotation data for field pea and faba bean ESTs, with totals of 22,057 and 18,052 unique hits, respectively. These values are very close to the estimated number of total genes (c. 25,000) present in a typical diploid plant genome, based on data from rice (*Oryza sativa *L.), sorghum (*Sorghum bicolor *L.), *A. thaliana *and *Brachpodium distachyon *[49,50]. On this basis, the sequences annotated in this study are likely to represent c. 88% and c. 72% of the gene complements of field pea and faba bean, respectively. Such estimates are also supported by comparison with the *M. truncatula *genome, from which a total of 11,737 unique hits obtained from field pea represented c. 49% of the known gene space, and 10,179 unique hits from faba bean represented c. 41% of the known gene space. Comparisons were also made to *G. max*, which is more distantly related to the Vicieae tribe species than *M. truncatula*, being located outside the Hologalegina clade, A total of 19,451 unique hits from field pea and 16, 497 from faba bean represent c. 35% and 30% of the known gene space respectively, based on total of predicted 55,787 protein-coding loci in the palaeopolyploid genome of soybean. In comparison to the genome of *A. thaliana*, which is more distantly related to both model and crop legume species within the dicotyledonous plants, the corresponding values were c. 25% for field pea and c. 20% for faba bean.

### Marker discovery and validation

One major advantage of second-generation DNA sequencing technologies is the capacity for computational interrogation of transcriptome data in order to develop large numbers of gene-based genetic markers such as SSRs and SNPs, of which few are currently available in the public domain for either field pea or faba bean. The EST-SSR primer pair sets generated in the current study will prove directly useful for the target species, and due to likely primer site conservation, may also be readily transferable to closely related species [51]. The transcriptome data generated in the current study, being derived from distinct genotypes, may potentially be also used for the detection of SNP markers in field pea and faba bean, to further enrich the available genomic resources for these two species.

The relative proportions of SSR array types in field pea and faba bean were similar to those observed in other plant species [8,52-54]. In theory, the frequencies of di-, tri-, tetra-, penta-, and hexanucleotide repeats should progressively decrease, based on the relative probability of replication slippage events. However, trinucleotide repeat units were predominant, followed by tetra-, di-, hexa-, and pentanucleotide repeat units. This observation is quite common for EST-derived SSRs, as trinucleotide expansions (or multiples thereof) within translated regions are capable of maintaining reading frame and hence generating a homopolymeric amino acid run within a partially or fully active protein.

The validation results for sub-sets of EST-SSR markers demonstrated that inclusion of non-domesticated genotypes in the study increased rates of polymorphism detection, consistent with the results of similar studies [8,55]. EST-SSRs generated in the present study will consequently provide a valuable tool for the understanding of global genetic diversity among both non-domesticated and cultivated pea and faba bean germplasm, as well as for dissection of the genetic control of important agronomic traits.

## Conclusions

In the current study, the generation of EST-datasets for field pea and faba bean has been described. Unigene sets obtained from field pea and faba bean were annotated against different genomic databases including those of *M. truncatula, A. thaliana, G. max*, and the nr database from GenBank. Furthermore, the EST dataset was used for design of EST-SSRs, subsets of which were validated across a number of cultivated and wild genotypes of pea and faba bean, indicating effectiveness of polymorphism detection and cross transferability.

## Authors' contributions

SK and LP contributed equally to the GS FLX sequencing, EST dataset analysis, EST-SSR marker assay design and interpretation of SSR genotyping data. SK and JF drafted the manuscript. KS assisted the sequence contig annotation process. NC contributed to data analysis, interpretation and assisted in drafting the manuscript. NC, SK, JF, TL, JP and MM co-conceptualised and coordinated the project. MM assisted in drafting the manuscript. All authors read and approved the final manuscript.

## Supplementary Material

Additional file 1 **Consensus sequences of assembled contigs from field pea**. The data represents the consensus sequences of 13,602 assembled contigs generated as a result of *de novo *assembly of field pea ESTs. Click here for file

Additional file 2 **Sequence information on singletons from field pea**. The data represents the sequence information on all the singletons generated from *de novo *assembly of field pea ESTs. Click here for file

Additional file 3 **Consensus sequences of assembled contigs from faba bean**. The data represents the consensus sequences of 6,370 assembled contigs generated as a result of *de novo *assembly of faba bean ESTs. Click here for file

Additional file 4 **Sequence information on singletons from faba bean**. The data represents the sequence information on all the singletons generated from *de novo *assembly of faba bean ESTs. Click here for file

Additional file 5 **Bioinformatic annotation (BLASTN) of field pea unigene set against the *Medicago truncatula *genome**. This file contains the BLAST results obtained as a result of comparison of field pea unigene set against the *M. truncatula *genome at an E value < 10^-10^. Click here for file

Additional file 6 **Bioinformatic annotation (BLASTX) of field pea unigene set against the nr database of GenBank**. This file contains the BLAST results obtained as a result of comparison of field pea unigene set against the GenBank nr database at an E value < 10^-10^. Click here for file

Additional file 7 **Bioinformatic annotation (BLASTN) of field pea unigene set against the *Arabidopsis thaliana *genome**. This file contains the BLAST results obtained as a result of comparison of field pea unigene set against the *A. thaliana *genome at an E value < 10^-10^. Click here for file

Additional file 8 **Bioinformatic annotation (BLASTN) of faba bean unigene set against the *Medicago truncatula *genome**. This file contains the BLAST results obtained as a result of comparison of faba bean unigene set against the *M. truncatula *genome at an E value < 10-10_._ Click here for file

Additional file 9 **Bioinformatic annotation (BLASTX) of faba bean unigene set against nr database of GenBank**. This file contains the BLAST results obtained as a result of comparison of faba bean unigene set against the GenBank nr database at an E value < 10^-10^. Click here for file

Additional file 10 **Bioinformatic annotation (BLASTN) of faba bean unigene set against *Arabidopsis thaliana *genome**. This file contains the BLAST results obtained as a result of comparison of faba bean unigene set against the *A. thaliana *genome at an E value < 10^-10^. Click here for file

Additional file 11 **Bioinformatic annotation (BLASTN) of field pea and faba bean unigene sets against the *Glycine max *genome**. This file contains the BLAST results obtained as a result of comparison of field pea and faba bean unigene sets against *G. max *genome at an E value < 10^-10^. Click here for file

Additional file 12 **Bioinformatic annotation (BLASTN) of field pea and faba bean unigene sets against the *Pisum sativum *transcriptome dataset from Franssen *et al *2011**. This file contains the BLAST results obtained as a result of comparison of field pea and faba bean unigene sets against *P. sativum *transcriptome dataset at an E value < 10^-10^. Click here for file

Additional file 13 **BLASTN of field pea and faba bean contigs and singltones against GenBank EST and nucleotide (nt) data**. This file contains the BLASTN results obtained as a result of comparison of field pea and faba bean contigs and singletons set against the EST and nucleotide (nt) databse of GenBank at an E value < 10^-10^. Click here for file

Additional file 14 **Sequence information of all of the SSR primer pairs identified and designed using BatchPrimer3 from field pea and faba bean ESTs**. This file contains all of the information (sequence information, orientation, sequence length, expected product length, Tm, GC content and SSR motif length) on SSR primer pairs designed using BatchPrimer 3. Click here for file

Additional file 15 **Characterisation of a sub-sets of EST-SSRs on wild and cultivated genotypes of field pea and faba bean**. This file represents the data on number and size of alleles amplified from screening of subsets of EST-SSRprimer pairs on different genotypes of field pea and faba bean. Click here for file
